# Establishment of early diagnosis models for cervical precancerous lesions using large-scale cervical cancer screening datasets

**DOI:** 10.1186/s12985-022-01908-w

**Published:** 2022-11-05

**Authors:** Bo Meng, Guibin Li, Zhengyu Zeng, Baowen Zheng, Yuyue Xia, Chen Li, Minyu Li, Hairong Wang, Yuelong Song, Shihui Yu

**Affiliations:** 1 Guangzhou KingMed Transformative Medicine Institute Co., Ltd., Guangzhou, Guangdong China; 2 Guangzhou KingMed Center for Clinical Laboratory Co., Ltd, Guangzhou, Guangdong China

**Keywords:** Human papillomavirus, Cervical cancer, Viral load, Logistic regression, Machine learning, Diagnostic model

## Abstract

**Background:**

Human papilloma virus (HPV) DNA test was applied in cervical cancer screening as an effective cancer prevention strategy. The viral load of HPV generated by different assays attracted increasing attention on its potential value in disease diagnosis and progression discovery.

**Methods:**

In this study, three HPV testing datasets were assessed and compared, including Hybrid Capture 2 (n = 31,954), Aptima HPV E6E7 (n = 3269) and HPV Cobas 4800 (n = 13,342). Logistic regression models for diagnosing early cervical lesions of the three datasets were established and compared. The best variable factor combination (VL + BV) and dataset (HC2) were used for the establishment of six machine learning models. Models were evaluated and compared, and the best-performed model was validated.

**Results:**

Our results show that viral load value was significantly correlated with cervical lesion stages in all three data sets. Viral Load and Bacterial Vaginosis were the best variable factor combination for logistic regression model establishment, and models based on the HC2 dataset performed best compared with the other two datasets. Machine learning method Xgboost generated the highest AUC value of models, which were 0.915, 0.9529, 0.9557, 0.9614 for diagnosing ASCUS higher, ASC-H higher, LSIL higher, and HSIL higher staged cervical lesions, indicating the acceptable accuracy of the selected diagnostic model.

**Conclusions:**

Our study demonstrates that HPV viral load and BV status were significantly associated with the early stages of cervical lesions. The best-performed models can serve as a useful tool to help diagnose cervical lesions early.

**Supplementary Information:**

The online version contains supplementary material available at 10.1186/s12985-022-01908-w.

## Background

Cervical cancer is the second most severe female cancer worldwide with 570,000 women diagnosed and 311,365 women died in the year 2018 despite worldwide applications of early screening for the disease or for the presence of human papillomavirus (HPV) [1]. It was estimated that 44.4 million cervical cancer cases would be diagnosed globally over the period of 2020–2069 [2]. Commonly used screening methods include HPV test, thin prep cytological test (TCT), and joined tests by HPV and TCT [3]. By comparison, TCT has lower false positive and higher false-negative rates than HPV test, but HPV test may cause higher unnecessary referrals to colposcopy [4]. With more and more HPV and TCT joined tests applied and compared [5–8], WHO changed cervical cancer screening guideline and listed HPV DNA test as the first recommended method for the application.

Currently, the results of HPV testing were generally reported as HPV positive or negative qualitatively based on the cut-off value of the assay used for the diagnosis. However, accumulated HPV screening data showed that HPV viral load could add valuable information as a screening triage marker. For example, Thomas identified a significant correlation between HPV viral load and integration status with high-grade squamous intraepithelial lesion (HSIL) [9]. Zhao’s study found that the 10-year cumulative incidence rate of cervical intraepithelial neoplasia (CIN2 +) was associated with cytological lesions and viral load and they recommended viral loads as a triage marker for non-16/18 hrHPV (high risk HPV) positive women [10]. A recent study also indicated that HPV viral load was positively correlated with cervical lesion grade based on 8556 women’s cervical cancer screening results [11]. In addition to being considered as a potential triage marker, HPV viral load was also a potential disease progression indicator as being showed that cervical cancer patients with high HPV viral load had a significantly lower 15-year survival rate and an advanced stage based on the International Federation of Gynaecology and Obstetrics (FIGO) as well as increased recurrence rate [12]. However, inconsistent conclusions related to viral load triage and prediction value from different studies restrain applications of viral load value in clinical settings [13]. One of the reasons causing result inconsistency is likely due to the different methods used in different diagnostic laboratories as being shown by a few small sizes of HPV viral load studies based on Hybrid Capture 2 (HC2) [14], Aptima E6E7 [15], and Cobas 4800 [16].

In this study, we retrospectively compared our cervical cancer screening results assayed by the 3 HPV testing platforms (HC2, Aptima E6E7, and HPV Cobas 4800) with accompanied TCT test results. A model for predicting different levels of cervical lesions was established by integrating potential cervical cancer risk factors, such as HPV infection status, HPV viral load, age, bacterial vaginosis, fungus, etc.

## Materials and methods

### Patients and data collection

**Fig. 1:**
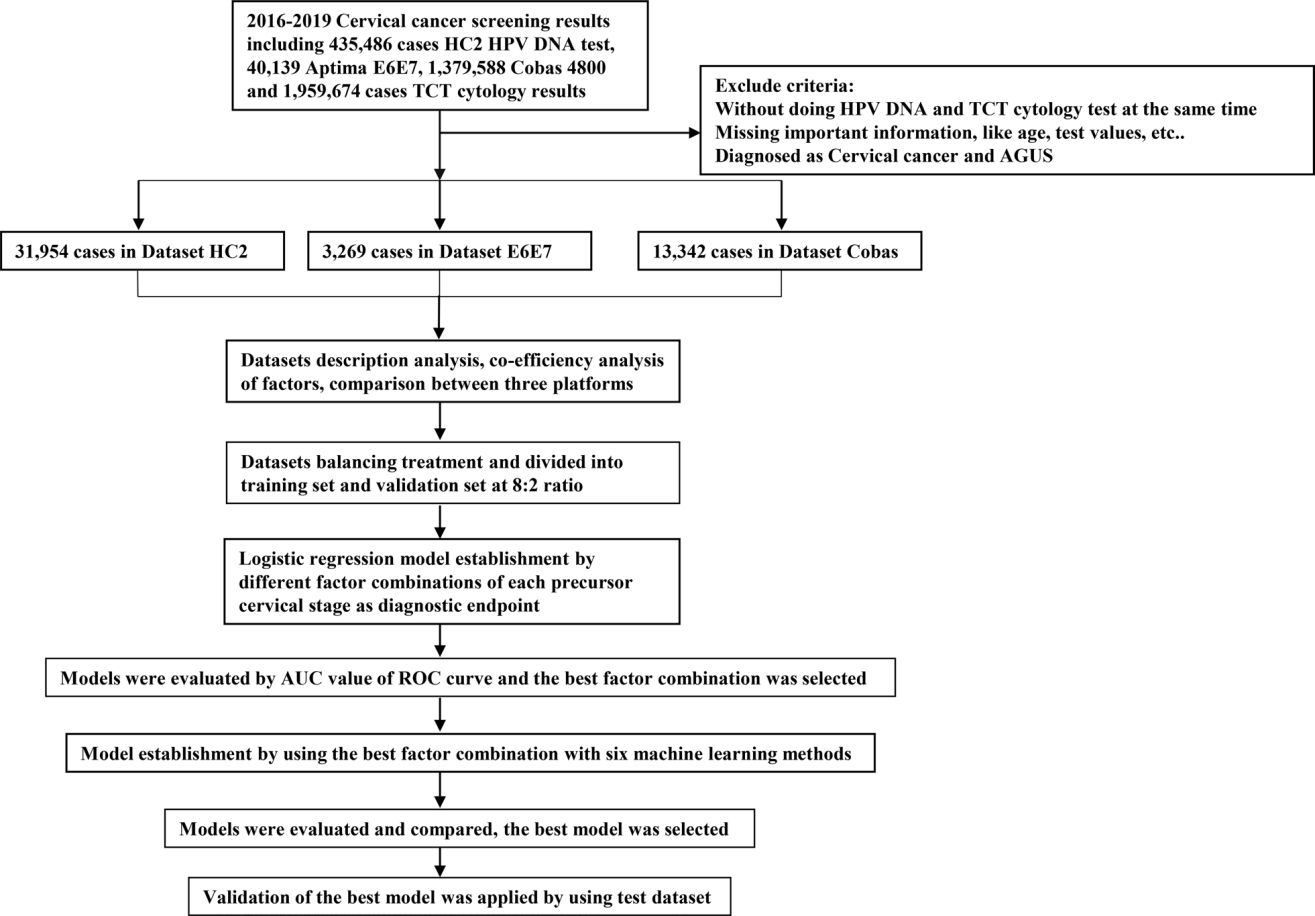
Flow chart diagram of study design and data analysis procedures

In total, 48,565 individuals were tested by both TCT and one of the 3 HPV testing methods (31,954 individuals tested by HC2, 3269 individuals tested by Aptima E6E7, and 13,342 individuals tested by Cobas 4800) from the years of 2016 to 2019 in our laboratory, a CAP- and ISO15189-accredited reference laboratory in Guangzhou, China. (Fig. 1). The cases were collected in three datasets, named Dataset HC2, Dataset E6E7, Dataset Cobas, respectively. The institutional review board of KingMed Diagnostics approved the study with code 022.

### HPV testing

HC2 assay detects 13 hrHPV subtypes, including HPV 16, 18, 31, 33, 35, 39, 45, 51, 52, 56, 58, 59 and 68 using hybrid Capture 2 high-Risk HPV DNA Test from Digene Corporation (Gaithersburg, MD, USA), providing an HPV positive or negative result based on the reading value compared with the cutoff value, RLU/CO > 1.0. Aptima HPV assay targets E6E7 mRNA expression of 14 hrHPV subtypes, including HPV 16, 18, 31, 33, 35, 39, 45, 51, 52, 56, 58, 59, 66 and 68 using TMA (transcription mediated amplification) based methodology from Hologic Company (Marlborough, MA, USA). Roche Cobas 4800 HPV DNA assay (Pleasanton, CA, USA) is a real-time PCR-based assay used for HPV16, HPV18, and other 12 hrHPV subtypes, including HPV31, 33, 35, 39, 45, 51, 52, 56, 58, 59, 66, and 68.

### TCT testing-liquid-based cytology

Collected specimens were automatically treated and converted to cytological specimens by using ThinPrep method from Hologic (Bedford, MA, USA) [17]. Prepared specimens were evaluated independently by at least 2 certified cyto-pathologists. Results were classified as: negative for intraepithelial lesion or malignancy (NILM); atypical squamous cells of undetermined significance (ASCUS); atypical squamous cell cannot exclude high-grade squamous intraepithelial lesion (ASC-H); low-grade squamous intraepithelial lesion (LSIL); high-grade squamous intraepithelial lesion (HSIL) [18]. Patients with a diagnosis of AGUS or cervical cancer were excluded from the study due to the limited number of individuals identified. Meanwhile, BV and fungal infections are determined by pathologists through the result of TCT.

### Data processing

Each of the 3 HPV platform datasets was divided into two datasets, all cases dataset (ACD), and dataset with only HPV positive cases (POS). HPV viral load values were calculated based on the reported value from each method, RLU/CO from HC2, S/CO from Aptima E6E7, and PCR cycle number from Cobas 4800.

### Risk factors selection and model establishment

The original datasets were divided into 2 datasets, the training dataset contained 80% of the cases while the validation dataset had 20%. Synthetic minority over-sampling technique (SMOTE) analysis using the DMwR package was applied to balance data before model establishment. Pearson's correlation coefficient was applied to determine the association between viral load, age, HPV infection status, BV, and fungus infection with cytology diagnostic stages (ASCUShigher, ASC-Hhigher, LSILhigher, HSILhigher). Different combinations of the significantly correlated variable factors were used for further logistic regression model analysis, and comparison was applied by using the area under curve (AUC) value of each receiver operating characteristic (ROC) curve. Besides logistic regression model analysis, five more machine learning methods, including Decision tree, Xgboost, Random forest, support vector machines (SVM), and Neural net, were applied to build models using the Rattle package with default parameters.

## Results

### Data sets characteristics and comparisons

All diagnostic results and related information were summarized in Table 1. In total, the average positive detection rate for HPV was 46.64% (22,654/48,565), including 59.10% (18,878/31,954) identified by HC2, 25.52% (3406/13,342) identified by Cobas 4800, and 11.31% (370/3269) identified by Aptima E6E7. Of the TCT results, NILM represented about 80% of the cases assayed, followed by LSIL (14%), ASCUS (7%), HSIL (3%), and ASC-H (2.6%). The proportions of cases with different TCT stages were similarly distributed among all 3 platform datasets (Additional file 1: Supplemental Fig. 1).

**Table 1:** Demographic data of patients collected in the three datasets

Patient status	HC2	E6E7	Cobas-all	Cobas-OT	Cobas-HPV16	Cobas-HPV18
*No. of women (percentage)*						
Total patient number	31,954	3269	13,342	13,342	13,342	13,342
HPV positive number	18,878 (59.1%)	370 (11.31%)	3406 (25.5%)	3229 (24.2%)	166 (1.24%)	95 (0.71%)
HPV negative number	13,076 (40.9%)	2899 (88.69%)	9936 (74.4%)	10,113 (75.8%)	13,176 (98.76%)	13,247 (99.29%)
NILM-ACD	25,876 (81%)	2955 (90.40%)	11,760 (88.1%)			
ASCUS-ACD	2871 (9.0%)	163 (4.98%)	824 (6.1%)			
ASC-H-ACD	492 (1.5%)	17 (0.52%)	88 (0.6%)			
LSIL-ACD	2039 (6.4%)	119 (3.64%)	572 (4.2%)			
HSIL-ACD	676 (2.1%)	15 (0.46%)	98 (0.7%)			
NILM-POS	13,319 (70.6%)	230 (62.16%)	2233 (65.4%)	2120 (65.6%)	88 (53.0%)	61 (64.2%)
ASCUS-POS	2416 (12.8%)	55 (14.86%)	481 (14.1%)	454 (14.1%)	19 (11.4%)	15 (15.8%)
ASC-H-POS	486 (2.6%)	11 (2.97%)	83 (2.4%)	74 (2.3%)	11 (6.6%)	4 (4.2%)
LSIL-POS	1981 (10.5%)	64 (17.3%)	517 (15.1%)	497 (15.4%)	25 (15.1%)	14 (14.7%)
HSIL-POS	676 (3.6%)	10 (2.7%)	97 (2.8%)	84 (2.6%)	23 (13.9%)	1 (1.1%)
ACD-BV	1095 (3.42%)	102 (3.12%)	450 (3.37%)	198 (1.48%)	9 (0.07%)	6 (0.04%)
ACD-Fungus	645 (2.01%)	129 (3.95%)	309 (2.32%)	82 (0.61%)	10 (0.07%)	4 (0.03%)
< 30 years (ACD)	1921 (6.01%)	634 (18.39%)	1230 (9.22%)			
≥ 30 years (ACD)	30,033 (93.99%)	2635 (80.61%)	12,112 (90.78%)			
< 30 years (POS)	234 (1.24%)	81 (21.9%)		149 (4.61%)	26 (15.7%)	23 (24.2%)
≥ 30 years (POS)	18,644 (98.8%)	289 (78.1%)		3080 (95.4%)	140 (84.3%)	72 (75.8%)
*Mean (SD) of HPV assay result values*						
Total patient number	142.159 (± 2.56)	1.188 (± 0.06)				
HPV positive number	240.494 (± 4.19)	10.424 (± 0.27)		33.117 (± 0.091)	30.143 (± 0.425)	32.045 (± 0.595)
HPV negative number	0.192 (± 0.001)	0.009 (± 0.0009)				
NILM-ACD	40.390 (± 1.22)	0.724 (± 0.05)				
ASCUS-ACD	273.412 (± 10.22)	3.985 (± 0.52)				
ASC-H-ACD	364.209 (± 23.34)	7.786 (± 1.67)				
LSIL-ACD	984.328 (± 23.21)	7.038 (± 0.70)				
HSIL-ACD	778.465 (± 29.84)	8.273 (± 1.66)				
NILM-POS	78.288 (± 2.32)	9.225 (± 0.30)		34.354 (± 0.10)	32.656 (± 0.54)	33.543 (± 0.61)
ASCUS-POS	324.864 (± 11.85)	11.767 (± 0.84)		32.762 (± 0.24)	30.253 (± 1.11)	31.2 (± 1.42)
ASC-H-POS	368.702 (± 23.56)	11.950 (± 1.42)		31.55 (± 0.56)	27.145 (± 0.66)	36.225 (± 1.96)
LSIL-POS	1013.138 (± 23.58)	13.003 (± 0.69)		28.876 (± 0.26)	26.368 (± 1.05)	25.429 (± 1.61)
HSIL-POS	778.465 (± 29.84)	12.409 (± 0.92)		30.281 (± 0.65)	25.974 (± 0.63)	29.3 (± NA)
ACD-BV	196.805 (± 15.59)	2.047 (± 0.44)				
ACD-Fungus	138.861 (± 18.14)	1.026 (± 0.28)				
< 30 years (ACD)	33.934 (± 5.9273)	1.379 (± 0.1648)				
≥ 30 years (ACD)	149.082 (± 2.6922)	1.142 (± 0.0708)				
< 30 years (POS)	277.189 (± 45.689)	10.717 (± 0.6574)		31.554 (± 0.4467)	30.612 (± 1.0022)	31.117 (± 1.352)
≥ 30 years (POS)	240.034 (± 4.200)	10.341 (± 0.2959)		33.192 (± 0.0935)	30.056 (± 0.4699)	32.342 (± 0.657)
*Mean (SD) of age*						
Total patient	44.901 (± 0.0561)	37.597 (± 0.1633)	42.793 (± 0.0840)	45.808 (± 0.1686)	39.012 (± 0.7513)	38.284 (± 1.0675)

*ACD* all cases dataset result, *POS* positive dataset result, *Cobas-OT* results of other 12 high risk HPV types in Cobas dataset, *Cobas-all* all the HPV types in Cobas dataset, *Cobas-HPV16* HPV16 result in Cobas dataset, *Cobas-HPV18* HPV18 result in Cobas dataset

**Fig. 2:**
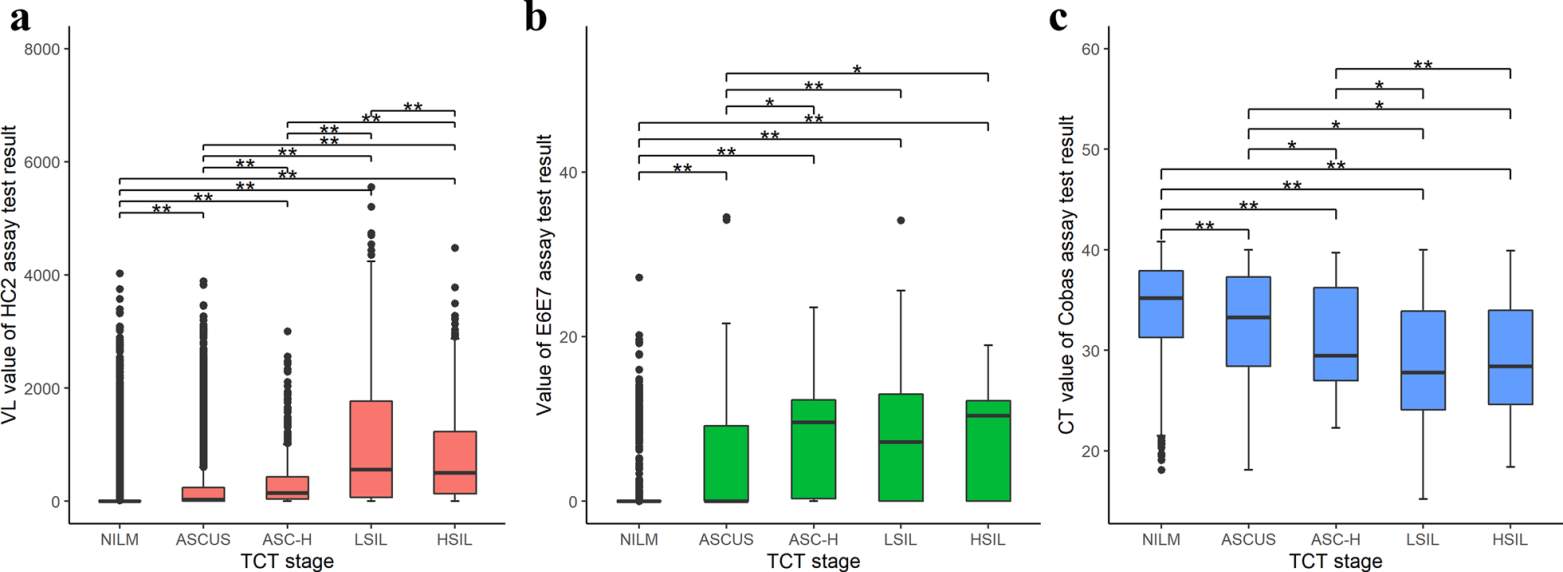
Distribution of viral load value with cervical lesion stages of the three platform ACDs. **a** HC2. **b** E6E7. **c** Cobas

The viral loads showed an increasing trend along with the advancing cytology stages in each of the 3 HPV datasets (Fig. 2 and Additional file 1: Supplemental Fig. 2). Viral load values of each two stages were found significantly different in HC2 ACD except that between stage ASCUS and ASC-H. Compared with the other two platform datasets, more significant differences between TCT stages in the HC2 dataset were observed, no matter in ACD or positive dataset. Ct value of Cobas assay was used as viral load value and three types of HPV positive cases of Cobas were shown separately, other type HPV (HPV OT), HPV16, and HPV18.

### Correlations between variable factors

Correlation analysis was carried out to analyse the relationship among any 2 of the following factors (Additional file 1: Supplemental Table 2). In detail, we observed the following relations: (1) A significant correlation between viral load with cervical lesion stages in all the 3 datasets; (2) A significant correlation between age with cervical lesion stages in HC2 and Cobas datasets; (3) A significant correlation between viral load with BV infection in HC2 ACD, and E6E7 ACD but not in the POS of E6E7 and Cobas; (4) Fungus infection was observed significantly correlated with age but not with viral load and BV in all the three platform datasets; (5) There was no significant correlation between BV and age in most datasets, except HC2 POS. The detailed results were shown in Additional file 1: Supplemental Tables 2 and 3.

**Table 2:** Performance summary of models established by six ML methods in terms of PPV, NPV, Sensitivity, Specificity, Accuracy, Precision

	ACD							POS							P Value (ACD vs.POS)		
	AUC	95% CI	PPV	NPV	Sensitivity	Specificity	Accuracy	AUC	95% CI	PPV	NPV	Sensitivity	Specificity	Accuracy	AUC	PPV	NPV
*Decision tree*																	
ASCUS higher	0.884	0.877–0.8902	0.826	0.825	0.828	0.822	0.825	0.831	0.8211–0.8401	0.814	0.742	0.974	0.775	0.875	0.249	0.046	0.004
ASC-H higher	0.856	0.8499–0.8622	0.822	0.921	0.941	0.771	0.861	0.893	0.8853–0.9	0.799	0.860	0.902	0.726	0.822			
LSIL higher	0.881	0.8745–0.8864	0.831	0.943	0.957	0.785	0.875	0.824	0.815–0.8321	0.795	0.888	0.924	0.718	0.875			
HSIL higher	0.875	0.8692–0.8799	0.813	0.967	0.974	0.775	0.875	0.814	0.8064–0.8224	0.755	0.919	0.940	0.688	0.816			
*Xgboost*																	
ASCUS higher	0.915	0.9095–0.9205	0.838	0.826	0.826	0.838	0.832	0.860	0.8512–0.8687	0.815	0.750	0.725	0.834	0.779	0.005	0.005	< 0.001
ASC-H higher	0.953	0.9494–0.9563	0.869	0.897	0.914	0.845	0.881	0.910	0.9037–0.917	0.829	0.822	0.858	0.788	0.826			
LSIL higher	0.956	0.9523–0.9591	0.871	0.911	0.925	0.849	0.889	0.924	0.918–0.9291	0.841	0.833	0.863	0.807	0.779			
HSIL higher	0.961	0.9587–0.9642	0.856	0.946	0.952	0.838	0.896	0.929	0.9237–0.934	0.814	0.874	0.889	0.793	0.841			
*Random forest*																	
ASCUS higher	0.896	0.8895–0.9021	0.834	0.822	0.822	0.834	0.828	0.845	0.8354–0.8538	0.814	0.742	0.712	0.835	0.773	0.004	0.026	0.002
ASC-H higher	0.935	0.9304–0.9389	0.872	0.895	0.912	0.849	0.882	0.900	0.8926–0.9069	0.820	0.831	0.870	0.769	0.824			
LSIL higher	0.938	0.9336–0.9422	0.868	0.914	0.927	0.845	0.888	0.907	0.9003–0.9131	0.823	0.849	0.884	0.776	0.773			
HSIL higher	0.952	0.9485–0.955	0.845	0.952	0.959	0.823	0.891	0.920	0.9139–0.9253	0.825	0.857	0.868	0.811	0.840			
*SVM*																	
ASCUS higher	0.904	0.898–0.9099	0.904	0.727	0.656	0.929	0.791	0.852	0.8426–0.8608	0.883	0.796	0.771	0.897	0.834	0.003	0.045	0.449
ASC-H higher	0.943	0.9386–0.9467	0.917	0.771	0.756	0.923	0.835	0.901	0.8932–0.9077	0.899	0.705	0.684	0.908	0.786			
LSIL higher	0.947	0.9425–0.9504	0.910	0.787	0.775	0.916	0.842	0.915	0.9088–0.9209	0.904	0.729	0.715	0.910	0.834			
HSIL higher	0.932	0.9272–0.9365	0.883	0.796	0.771	0.897	0.834	0.896	0.889–0.9032	0.848	0.736	0.695	0.872	0.783			
*Logistic regression*																	
ASCUS higher	0.905	0.8987–0.9105	0.906	0.725	0.652	0.931	0.790	0.852	0.8426–0.8608	0.872	0.690	0.595	0.912	0.753	0.005	0.027	0.004
ASC-H higher	0.943	0.9393–0.9473	0.920	0.762	0.743	0.927	0.829	0.901	0.8942–0.9086	0.898	0.706	0.686	0.906	0.786			
LSIL higher	0.947	0.9428–0.9506	0.913	0.779	0.763	0.920	0.838	0.916	0.9097–0.9217	0.904	0.729	0.715	0.910	0.753			
HSIL higher	0.934	0.9291–0.938	0.888	0.778	0.744	0.905	0.824	0.903	0.8961–0.9093	0.851	0.712	0.651	0.883	0.766			
*Neural net*																	
ASCUS higher	0.909	0.903–0.9145	0.832	0.826	0.828	0.830	0.829	0.854	0.8449–0.8629	0.792	0.757	0.746	0.802	0.774	0.004	0.003	< 0.001
ASC-H higher	0.951	0.9471–0.9542	0.868	0.901	0.918	0.843	0.883	0.908	0.9015–0.915	0.816	0.834	0.874	0.764	0.824			
LSIL higher	0.954	0.9501–0.9572	0.866	0.915	0.928	0.842	0.887	0.919	0.9135–0.9252	0.837	0.837	0.868	0.800	0.774			
HSIL higher	0.959	0.9561–0.962	0.855	0.945	0.951	0.838	0.895	0.926	0.9203–0.931	0.813	0.874	0.888	0.791	0.840

**Table 3:** AUC value of the best two models established by Xgboost with test dataset analysis

Data sets	Accuracy	ASCUShigher	ASC-Hhigher	LSILhigher	HSILhigher	*P* value (ACD vs. POS)
ACD	xgboost-AUC	0.8200	0.9385	0.9413	0.9293	0.00050
	Sensitivity	0.5020	0.6476	0.6484	0.6000	
	Specificity	0.9547	0.9561	0.9577	0.9568	
	Accuracy	0.9274	0.9484	0.951	0.9556	
POS	xgboost-AUC	0.7176	0.7285	0.7210	0.7336	
	Sensitivity	0.5747	0.6596	0.5750	0.6429	
	Specificity	0.7466	0.6907	0.7199	0.7228	
	Accuracy	0.6909	0.6853	0.6983	0.7207	

### Logistic regression models build on different factor combinations

**Fig. 3:**
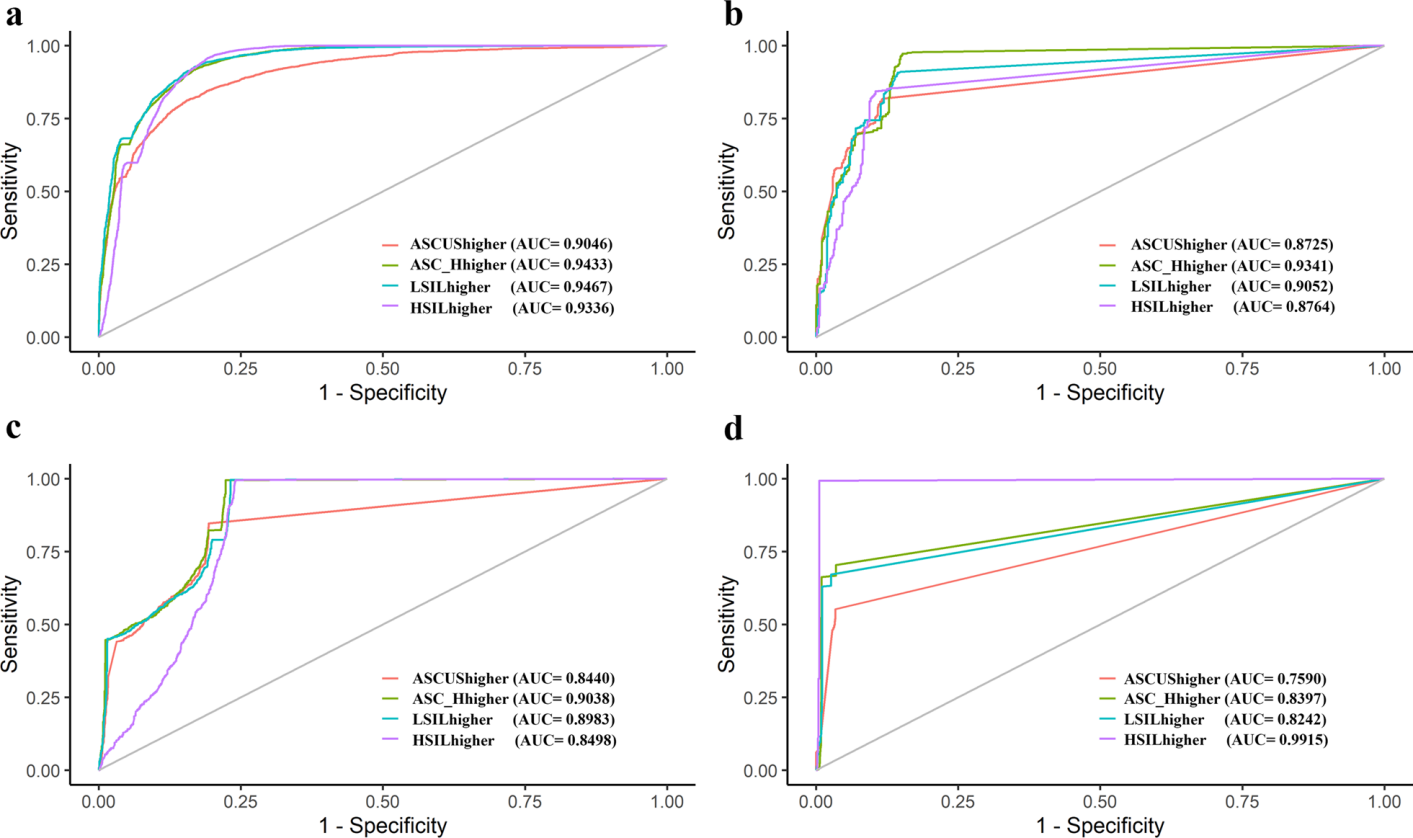
ROC curve of logistic regression model established by VL and BV variables using all data sets of the three platforms. **a** HC2. **b** E6E7. **c** Cobas_OT. **d** Cobas_16

The logistic regression model of each test dataset was established with every precancerous stage and higher as a diagnostic endpoint. Different risk factor combinations of viral load, BV, and age were used for building the regression equation. The AUC value of each model and comparison results of each two-variable combinations were summarized in Additional file 1: Supplemental Table. To avoid data imbalance, SMOTE was applied to balance the data of each cervical lesion stages. The results, elucidated that: (1) models of HC2 ACD and POS all performed best compared with the models established by the other two platform data sets with significant difference (Additional file 1: Supplemental Table 5); (2) models of HC2 POS and ACD with HPV viral load and bacterial vaginosis as variables performed best with significant difference compared with models established by viral load (VL) only and VL with Age variables (Additional file 1: Supplemental Table 6). ROC curves of each platform ACD models were shown in Fig. 3. It showed that models performed differently by using different cervical lesion stages and higher as a diagnostic endpoint. Models of HC2 performed best (AUC = 0.9467) with LSIL higher stage as a diagnostic endpoint. E6E7 (AUC = 0.9341) and Cobas OT models (AUC = 0.9038) performed best with ASC-H higher stage as a diagnostic endpoint. However, Cobas 16 models performed best (AUC = 0.9915) with HSIL higher stage as a diagnostic endpoint. In summary, the models generated by the HC2 platform with BV and VL as variables had the best performance compared with models of the other two platform data sets.

### Establishment and comparison of machine learning models

**Fig. 4:**
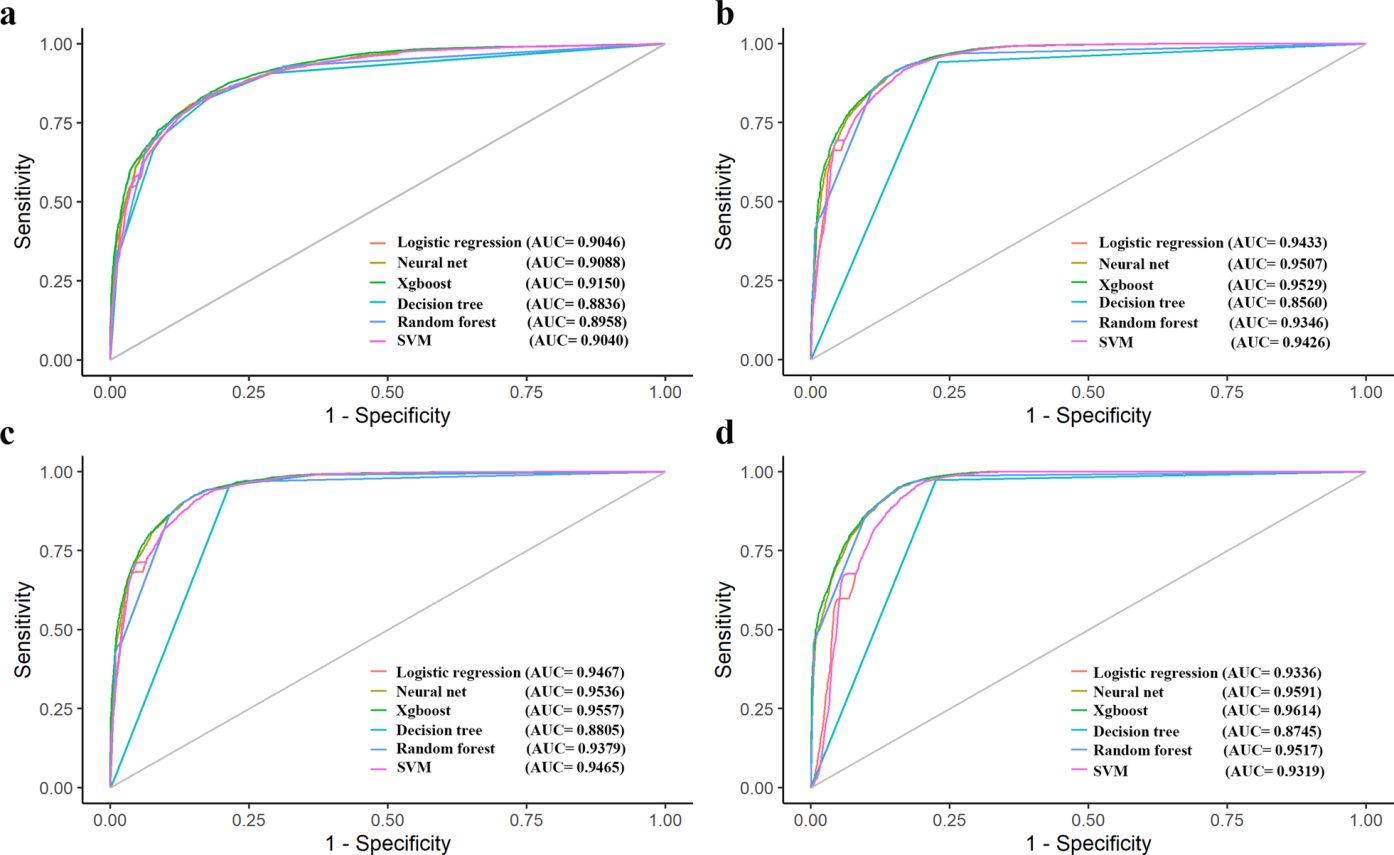
ROC curve of six machine learning methods model by using HC2 dataset. **a** ASCUS higher. **b** ASC-H higher. **c** LSIL higher. **d** HSIL higher

To establish the best model for diagnosing early cervical lesion stages, six machine learning methods were further applied in HC2 ACD and POS with VL and BV as variable factors. AUC values, PPV, NPV, accuracy, sensitivity, and specificity of the models were analysed for model performance evaluation, shown in Table 2, and comparisons were carried out between different methods, Additional file 1: Supplemental Table 7. The results indicated that the AUC value of Xgboost models in both ACD and POS was the highest compared with the other five methods, with an AUC value of ASCUS higher, ASC-H higher, LSIL higher, and HISL higher were 0.915, 0.953, 0.956, and 0.961 in ACD and 0.860, 0.910, 0.924 and 0.929 in POS, respectively. The ROC curve of Xgboost models of each diagnostic endpoint were shown in Fig. 4. And a significant difference was observed between ACD and POS AUC values. The Xgboost models were evaluated with a sensitivity of 0.826 (ASCUS higher), 0.914 (ASC-H higher), 0.925 (LSIL higher) and 0.952 (HSIL higher) and specificity of 0.838 (ASCUS higher), 0.845 (ASC-H higher), 0.849 (LSIL higher) and 0.838 (HSIL higher) in HC2 ACD, respectively. The sensitivity and specificity of Xgboost models of HC2 POS were significantly lower (sensitivity, P = 0.007; specificity, P = 0.05) than them in ACD.

### Validation of the best HC2 models

To further validate the model established by Xgboost, we collected a new batch of HC2 HPV testing data, which consisted of 3932 NILM, 148 ASCUS, 28 ASC-H, 62 LISL, and 15 HSIL patients and evaluated the performance of the models in all and positive datasets. The results were summarized in Table 3. It showed that by using a new set of HC2 results, diagnostic models of Xgboost could predict the cytologic stage of the patient with acceptable AUC values, 0.8200 for ASCUS higher, 0.9385 for ASC-H higher, 0.9413 for LSIL higher, and 0.9293 for HSIL higher stage of test ACD model and 0.7176 for ASCUS higher, 0.7285 for ASC-H higher, 0.7210 for LSIL higher, and 0.7336 for HSIL higher stage of test positive data set. The ACD model performed better than the positive dataset with specificity ranging from 0.9547 to 0.9577 and sensitivity ranging from 0.5020 to 0.6484.

## Discussion

The mean values of HPV VL in each cytology stage increased with the severity of cervical lesion grade, consistent with previous findings, indicating the reliability of our conclusion [10, 19]. However, the associations of HPV subtypes VL with cervical lesions were inconsistent across studies. Luo Hongxue reported that the viral load of HPV16/18 could be used as a triage marker for HPV-positive women while Dong Li’s research found it cannot [10, 14]. The disagreement of studies might be caused by methods limitations in the studies or the reality of different viral load distribution characteristics of each HPV subtype in different populations. Based on our comparison results of platforms, which was seldom to be seen in one study, although the VL value trend seems similar among platforms, there was still a difference that could be observed in the distribution of viral load in each specific disease stage and coefficient among factors. It indicated that different methods could provide different detection ranges, which further differently reflected the real viral load situation of the sample. Therefore, the method with more broad detection range and lower limit of detection should be recommended for viral load study.

The cervical microbiome has been found to be affected by HPV infection [20] and the presence of BV was reported to be associated with HPV infection and persistence [21, 22]. BV and other factor, multiple sexual partners, were combined to predict of CIN/CC status [23]. A significant association between BV with HSIL cytologic stage in our HC2 dataset was observed, consistent with a previous report [24, 25]. These results provided strong support for our model comparison results which indicated that BV and VL are the two factors that provide the best accuracy for the effect of models. Although the BV status of our results was retrieved from cytologic diagnosis results, it also indicated the potential of DNA test assays or tools of detecting the two factors at the same time and collected information that could be used for cervical lesion prediction. The simultaneous detection method of HPV infection and microbiome of cervical samples have been developed by another study [26], providing the value of detecting both factors in the prevention of cervical cancer development. Since there were many factors that could affect cervical cancer development and their correlation relationship was not fully understood. Therefore, more exploration between them is necessary. The correlation analysis of risk factors in our study discovered a more significant correlation between them in specific population groups, which indicated different models with specific different factors might be established in the future to get more accurate results for clinical application.

Of the 3 HPV test platforms, Cobas 4800 is the only platform that could differentiate HPV16, HPV18, and HPV OT, enabling us to analyse the correlations between viral loads of the HPV subtypes and the severity of the cervical lesions caused by HPV. Our results showed that viral load in the cases with HPV16 infection increased more obviously with advanced cervical lesion stages compared with HPV18 and HPV OT, like a previous report [27]. If actual correlations between viral loads of HPV subtypes and cervical lesions caused by these viruses could be demonstrated, it might be possible to accurately diagnose people with similar conditions, using viral load and other variable factors without being necessarily referred to pathologists in the future [28].

This study indicated that: (1) HPV viral load values generated by the HC2 platform fit more for the diagnostic model establishment than the other two platforms, Aptima E6E7 and Cobas; (2) Sample balance treatment (SMOTE) improved our model performance in the unbalanced dataset since our datasets were from cervical cancer screening with a significantly higher percentage of normal status samples than abnormal samples. Similar results were reported showing that datasets pre-processed by SMOTE could improve model accuracy by avoiding bias caused by imbalance of the datasets used [29]. The AUC values of other diagnostic models had been reported as 0.895 and 0.64 in diagnosing CIN2 + by Tuerxun’s study and Xiao’s study, respectively [30, 31]. However, the AUC value of our model for HSIL prediction is 0.9293.

In summary, our results provided valuable information for the evaluation of viral load of HPV in clinical diagnostic applications. We also proved it is feasible to predict the cytological stage by using a diagnostic model based on viral load and other factors, especially in areas lacking enough pathological resources. As we all know that cervical cancer mainly occurs in low-level income countries, which often lack high-quality clinical resources, including clinicians and equipment. Therefore, our model with accurate diagnostic prediction function provides strong evidence for its clinical application with reliable results. However, due to the significant difference between HPV test methods, more studies need to be carried out to standardize the best way of diagnosing by models. Based on our study, the PCR-free method might be a better choice in this scenario. What’s more, further study combing patients’ information, cervical cancer screening results, colposcopy diagnose results, and management information should be carried out in the future to evaluate the application value of our model.

## Conclusions

Using clinical laboratory cervical cancer screening datasets, after evaluating optimal datasets, machine learning method, and variable factors, early diagnostic models of four cervical lesion stages were defined. It is the first study by using BV and HPV VL for cervical lesion cytological diagnosis prediction and the accuracy of the prediction was shown to be superior to other clinical characteristics. Furthermore, machine learning models built based on HPV VL and BV demonstrated excellent performance in determining cervical cancer precancerous lesions at different stages, especially the Xgboost model. These promising findings warrant the early diagnosis for cervical lesions in clinical applications, especially in scenarios with limited pathological resources.

## Supplementary Information

**Additional file 1.** Other details of this study.

## Data Availability

Due to the privacy of patients, the related data cannot be available for public access but can be obtained from Shihui Yu, Bo Meng upon reasonable request.

## Abbreviations

HPV: Human papillomavirus
HC2: Hybrid Capture 2
TCT: Thin prep cytologic test
BV: Bacterial vaginosis
VL: Viral load
ACD: All cases dataset
POS: HPV positive cases dataset
TMA: Transcription mediated amplification
AUC: Area under the curve
ROC: Receiver operating characteristic
PPV: Positive prediction value
NPV: Negative prediction value
SVM: Support vector machines
Xgboost: Extreme gradient boosting
SMOTE: Synthetic minority over-sampling technique
NILM: Negative for intraepithelial lesion or malignancy
ASCUS: Atypical squamous cells of undetermined significance
ASC-H: Atypical squamous cell cannot exclude high-grade squamous intraepithelial lesion
LSIL: Low-grade squamous intraepithelial lesion
HSIL: High-grade squamous intraepithelial lesion
