# Supplementary material for: Establishment of early diagnosis models for cervical precancerous lesions using large-scale cervical cancer screening datasets
Source: Virol J. 2022 Nov 5;19:177. doi: 10.1186/s12985-022-01908-w (PMC9636682; doi:10.1186/s12985-022-01908-w)

Supplemental Table 1. Mean value comparasion of over and less 30 year-old patient from the three platform datasets.

	No. of women		Mean value		P value
	<30	>=30	<30	>=30	(<30 vs >= 30)
HC2POS	234	18644	277.189	240.034	
HC2ACD	1921	30033	33.934	149.082	<0.01
E6E7POS	81	289	10.717	10.341	
E6E7ACD	634	2635	1.379	1.142	
Cobas-OTPOS	149	3080	31.554	33.192	<0.01
Cobas-16POS	26	140	30.612	30.056	
Cobas-18POS	23	72	31.117	32.342	

Note: ACD: all cases dataset; POS: positive cases dataset

Supplemental Table 2. Summary of correlation relationship analysis results of multiple factors.

	HC2				E6E7				Cobas					
	Age	Viral Load	BV	Fungus	Age	Viral Load	BV	Fungus	Age	HPV 16	HPV18	HPV OT	BV	Fungus
ASCUS higher	YACD-YPOS	YACD-YPOS	NACD-NPOS	NACD-NPOS	NACD-NPOS	YACD-YPOS	NACD-NPOS	NACD-NPOS	YACD-YPOS	YACD-YPOS	YACD-YPOS	YACD-YPOS	NACD-YPOS	NACD-YPOS
ASC-H higher	YACD-YPOS	YACD-YPOS	NACD-NPOS	NACD-NPOS	NACD-NPOS	YACD-YPOS			NACD-YPOS	YACD-YPOS	YACD-YPOS	YACD-YPOS		
LSIL higher	YACD-YPOS	YACD-YPOS	NACD-NPOS	NACD-NPOS	YACD-NPOS	YACD-YPOS			NACD-YPOS	YACD-YPOS	YACD-YPOS	YACD-YPOS		
HSIL higher	YACD-YPOS	YACD-YPOS	YACD-YPOS	NACD-NPOS	NACD-NPOS	YACD-NPOS			YACD-NPOS	NACD-NPOS	YACD-NPOS	YACD-YPOS		
TCT	YACD-YPOS	YACD-YPOS	YACD-YPOS	NACD-NPOS	NACD-NPOS	YACD-YPOS			YACD-YPOS	YACD-YPOS	YACD-YPOS	YACD-YPOS		
BV	NACD-YPOS	YACD-YPOS		NACD-NPOS	NACD-NPOS	YACD-NPOS		NACD-NPOS	NACD-NPOS	NACD-NPOS	NACD-NPOS	NACD-NPOS		NACD-NPOS
Fungus	YACD-YPOS	NACD-NPOS	NACD-NPOS		YACD-YPOS	NACD-NPOS	NACD-NPOS		YACD-YPOS	NACD-NPOS	NACD-NPOS	NACD-NPOS	NACD-NPOS	
Age		YACD-YPOS	NACD-YPOS	YACD-YPOS		NACD-NPOS	NACD-NPOS	YACD-YPOS		NACD-NPOS	NACD-NPOS	YACD-YPOS	NACD-NPOS	YACD-YPOS
Viral Load	YACD-YPOS		YACD-YPOS	NACD-NPOS	NACD-NPOS		YACD-NPOS	NACD-NPOS						
HPV infection status	YACD-NONE	YACD-NONE	YACD-NONE	YACD-NONE	NACD-NONE		NACD-NONE	NACD-NONE	YACD-NONE				YACD-NONE	NACD-NONE

Note: ACD: all cases dataset; POS: positive cases dataset; Y: correlation relationship significant; N: correlation relationship not significant; blank: results not available.

Supplemental Table 3. Correlation analysis result summary.

		HC2							
		VL		Age		BV		Fungus	
		r	p-value	r	p-value	X-squared	p-value	X-squared	p-value
VL	ACD	1.0000	NA	0.0370	0.0000	NP	NP	NP	NP
	POS	1.0000	NA	-0.1050	0.0000	NP	NP	NP	NP
Age	ACD	0.0370	0.0000	1.0000	NA	NP	NP	NP	NP
	POS	-0.1050	0.0000	1.0000	NA	NP	NP	NP	NP
hpv	ACD	0.2580	0.0000	0.4050	0.0000	39.1848	0.0000	17.0622	0.0000
	POS	NA	NA	NA	NA	NA	NA	NA	NA
BV	ACD	0.0220	0.0001	-0.0044	0.4335	NA	NA	0.2114	0.6456
	POS	0.0170	0.0202	-0.0450	0.0000	NA	NA	0.3437	0.5577
Fungus	ACD	-0.0010	0.8533	-0.0830	0.0000	0.2114	0.6456	NA	NA
	POS	0.0071	0.3274	-0.0900	0.0000	0.3437	0.5577	NA	NA
TCT	ACD	0.4810	0.0000	0.0510	0.0000	17.9508	0.0013	4.2851	0.3688
	POS	0.5000	0.0000	-0.1430	0.0000	18.5367	0.0010	0.8928	0.9256
ASCUShigher	ACD	0.4590	0.0000	0.0490	0.0000	0.5799	0.4463	3.9811	0.0460
	POS	0.4370	0.0000	-0.1370	0.0000	0.6667	0.4142	0.2588	0.6110
ASC-Hhigher	ACD	0.5140	0.0000	0.0350	0.0000	0.1591	0.6900	2.8418	0.0918
	POS	0.4840	0.0000	-0.1150	0.0000	3.3888	0.0656	0.3453	0.5568
LSILhigher	ACD	0.5270	0.0000	0.0110	0.0443	0.1910	0.6621	1.9558	0.1620
	POS	0.5020	0.0000	-0.1360	0.0000	0.7631	0.3823	0.0533	0.8174
HSILhigher	ACD	0.2040	0.0000	0.0360	0.0000	7.9149	0.0049	0.5347	0.4646
	POS	0.1800	0.0000	-0.0200	0.0058	11.3253	0.0008	0.0596	0.8071

		E6E7							
		VL		Age		BV		Fungus	
		r	p-value	r	p-value	X-squared	p-value	X-squared	p-value
VL	ACD	1.0000	NA	-0.0283	0.1057	NP	NP	NP	NP
	POS	1.0000	NA	-0.0428	0.4112	NP	NP	NP	NP
Age	ACD	-0.0283	0.1057	1.0000	NA	NP	NP	NP	NP
	POS	-0.0428	0.4112	1.0000	NA	NP	NP	NP	NP
hpv	ACD	0.8820	0.0000	-0.0241	0.1688	5.8303	0.0158	0.1853	0.6669
	POS	NA	NA	NA	NA	NA	NA	NA	NA
BV	ACD	0.0410	0.0197	0.0093	0.5967	NA	NA	2.3708	0.1236
	POS	0.0120	0.8185	-0.0233	0.6550	NA	NA	0.9052	0.3414
Fungus	ACD	-0.0089	0.6090	-0.0720	0.0000	2.3708	0.1236	NA	NA
	POS	-0.0929	0.0742	-0.1440	0.0056	0.9052	0.3414	NA	NA
TCT	ACD	0.3790	0.0000	-0.0115	0.5101	NP	NP	NP	NP
	POS	0.3020	0.0000	0.0558	0.2848	NP	NP	NP	NP
ASCUShigher	ACD	0.3800	0.0000	-0.0148	0.3966	3.3034	0.0691	0.1570	0.6919
	POS	0.2940	0.0000	0.0499	0.3383	0.1551	0.6937	0.0008	0.9773
ASC-Hhigher	ACD	0.3560	0.0000	-0.0203	0.2457	NP	NP	NP	NP
	POS	0.2480	0.0000	0.0577	0.2683	NP	NP	NP	NP
LSILhigher	ACD	0.3310	0.0000	-0.0380	0.0305	NP	NP	NP	NP
	POS	0.2390	0.0000	-0.0039	0.9401	NP	NP	NP	NP
HSILhigher	ACD	0.1280	0.0000	0.0029	0.8669	NP	NP	NP	NP
	POS	0.0634	0.2241	0.0126	0.8086	NP	NP	NP	NP

		Cobas											
		hpv12_value		hpv16_value		hpv18_value		Age		BV		Fungus	
		r	p-value	r	p-value	r	p-value	r	p-value	X-squared	p-value	X-squared	p-value
VL	ACD	NA	NA	NA	NA	NA	NA	NA	NA	NP	NP	NP	NP
	POS	NA	NA	NA	NA	NA	NA	NA	NA	NP	NP	NP	NP
Age	ACD	0.0520	0.0032	0.0256	0.7425	0.0187	0.8566	1.0000	NA	NP	NP	NP	NP
	POS	0.0520	0.0032	0.0254	0.7457	0.0447	0.6672	1.0000	NA	NP	NP	NP	NP
hpv	ACD	NA	NA	NA	NA	NA	NA	0.1620	0.0000	104.4208	0.0000	1.7414	0.1870
	POS	NA	NA	NA	NA	NA	NA	NA	NA	NA	NA	NA	NA
BV	ACD	0.0033	0.8491	0.0324	0.6774	0.1415	0.1690	0.0152	0.0796	NA	NA	0.2055	0.6503
	POS	0.0033	0.8525	0.0325	0.6776	0.1398	0.1767	-0.0322	0.0602	NA	NA	0.4144	0.5197
Fungus	ACD	-0.0279	0.1127	-0.0437	0.5754	0.0408	0.6930	-0.0830	0.0000	0.2055	0.6503	NA	NA
	POS	-0.0280	0.1122	-0.0436	0.5772	0.0363	0.7271	-0.1210	0.0000	0.4144	0.5197	NA	NA
TCT	ACD	-0.2850	0.0000	-0.4000	0.0000	-0.3580	0.0003	0.0350	0.0001	NP	NP	NP	NP
	POS	-0.2850	0.0000	-0.4010	0.0000	-0.3350	0.0009	-0.0930	0.0000	NP	NP	NP	NP
ASCUShigher	ACD	-0.1970	0.0000	-0.2070	0.0073	-0.3230	0.0013	0.0340	0.0001	0.0028	0.9577	2.3658	0.1240
	POS	-0.1960	0.0000	-0.2080	0.0072	-0.3000	0.0032	-0.0800	0.0000	17.6665	0.0000	3.9326	0.0474
ASC-Hhigher	ACD	-0.2100	0.0000	-0.2330	0.0025	-0.3690	0.0002	0.0163	0.0601	NP	NP	NP	NP
	POS	-0.2100	0.0000	-0.2340	0.0024	-0.3320	0.0010	-0.0940	0.0000	NP	NP	NP	NP
LSILhigher	ACD	-0.2170	0.0000	-0.2170	0.0049	-0.3980	0.0001	0.0004	0.9644	NP	NP	NP	NP
	POS	-0.2170	0.0000	-0.2190	0.0046	-0.3580	0.0004	-0.1180	0.0000	NP	NP	NP	NP
HSILhigher	ACD	-0.0470	0.0078	-0.0263	0.7361	-0.2030	0.0473	0.0230	0.0082	NP	NP	NP	NP
	POS	-0.0460	0.0097	-0.0275	0.7248	-0.0081	0.9381	-0.0009	0.9579	NP	NP	NP	NP

Note: ACD: all cases dataset; POS: positive cases dataset;r: coefficiency; NA: not available; NP: not applicable

Supplemental Table 4. AUC value summary of all the regression models established by combing different factors of HC2 datasets.

Dataset	Platform	Endpoint	No. of NILM	No. of Case	No. of NILM (Smoted)	No. of Case (Smoted)	Factor (VL)	95%CI	Factor (VL+Age)	95%CI	Factor (VL+BV)	95%CI	Factor (VL+BV+Age)	95%CI	P value (ACD vs. POS)VL	P value (ACD vs. POS)VL+ Age	P value (ACD vs. POS) (VL+BV)	P value (ACD vs. POS)(VL+ BV+Age)
ACD	HC2	ASCUS higher	25876	6078	23704	24312	0.8773	0.8706-0.8841	0.823	0.8147-0.8312	0.9046	0.8987-0.9105	0.8723	0.8652-0.8794	0.0035	0.0117	0.0050	0.0245
		ASC-H higher	28747	3207	28863	32070	0.9278	0.9231-0.9324	0.894	0.8884-0.8995	0.9433	0.9393-0.9473	0.9215	0.9167-0.9263				
		LSIL higher	29239	2715	27150	29865	0.9331	0.9286-0.9376	0.9197	0.9147-0.9247	0.9467	0.9428-0.9506	0.9392	0.935-0.9435				
		HSIL higher	31278	676	33800	34476	0.918	0.9128-0.9233	0.8852	0.8793-0.891	0.9336	0.9291-0.938	0.9135	0.9086-0.9184				
	E6E7	ASCUS higher	2955	314	3454	3454	0.783	0.762-0.804	0.719	0.6926-0.7455	0.8725	0.8549-0.8901	0.8422	0.8212-0.8631	0.0083	0.0276	0.0079	0.0122
		ASC-H higher	3118	151	3020	3171	0.9209	0.9037-0.938	0.902	0.8837-0.9204	0.9341	0.9199-0.9482	0.924	0.9089-0.9391				
		LSIL higher	3135	134	2814	2948	0.8997	0.8806-0.9188	0.8848	0.8642-0.9054	0.9052	0.8876-0.9229	0.8931	0.8739-0.9123				
		HSIL higher	3254	15	3000	3015	0.8689	0.8486-0.8891	0.8745	0.8547-0.8942	0.8764	0.8568-0.8959	0.8819	0.8629-0.901				
	Cobas-OT	ASCUS higher	11647	1513	11650	12104	0.7473	0.734-0.7607	0.763	0.749-0.7771	0.844	0.8333-0.8546	0.8503	0.8394-0.8612	0.0721	0.0742	0.0339	0.0376
		ASC-H higher	12444	716	14320	15036	0.8423	0.8304-0.8543	0.8446	0.8328-0.8563	0.9038	0.896-0.9116	0.9058	0.8981-0.9135				
		LSIL higher	12523	637	13377	14014	0.836	0.8236-0.8485	0.8399	0.8278-0.852	0.8983	0.89-0.9066	0.9011	0.893-0.9092				
		HSIL higher	13075	85	15300	15385	0.845	0.834-0.8561	0.8455	0.8345-0.8565	0.8498	0.8391-0.8605	0.8503	0.8396-0.861				
	Cobas-16	ASCUS higher	9615	482	10604	10122	0.5709	0.5627-0.5791	0.6274	0.6105-0.6443	0.759	0.7475-0.7705	0.7867	0.7729-0.8004	0.7441	0.6739	0.1920	0.1383
		ASC-H higher	9977	120	10800	10920	0.8418	0.8317-0.8519	0.843	0.8303-0.8556	0.8397	0.8293-0.85	0.8429	0.8303-0.8555				
		LSIL higher	9993	104	9360	9464	0.8258	0.8146-0.8371	0.8288	0.8147-0.8429	0.8242	0.8129-0.8356	0.8315	0.8175-0.8454				
		HSIL higher	10073	24	4320	4344	0.9914	0.9861-0.9967	0.9905	0.9849-0.9961	0.9915	0.9863-0.9967	0.9906	0.9851-0.9961				
POS	HC2	ASCUS higher	13319	5559	16677	16677	0.8108	0.8007-0.821	0.7628	0.7514-0.7741	0.8517	0.8426-0.8608	0.8251	0.8152-0.8351				
		ASC-H higher	15735	3143	15715	18858	0.8714	0.8631-0.8798	0.824	0.8143-0.8337	0.9014	0.8942-0.9086	0.8753	0.8671-0.8835				
		LSIL higher	16221	2657	18599	21256	0.897	0.8765-0.8911	0.8463	0.838-0.8547	0.9157	0.9097-0.9217	0.897	0.8902-0.9038				
		HSIL higher	18202	676	20280	20956	0.8637	0.8553-0.8722	0.8577	0.849-0.8663	0.9027	0.8961-0.9093	0.9028	0.8962-0.9093				
	E6E7	ASCUS higher	230	140	420	420	0.6732	0.5928-0.7536	0.6721	0.5914-0.7529	0.738	0.6632-0.8127	0.7399	0.6654-0.8145				
		ASC-H higher	285	85	425	510	0.6853	0.6066-0.7639	0.6814	0.6017-0.7612	0.7166	0.6404-0.7928	0.717	0.6405-0.7935				
		LSIL higher	296	74	518	592	0.6968	0.628-0.7657	0.7027	0.6339-0.7715	0.7605	0.6979-0.823	0.7629	0.7006-0.8253				
		HSIL higher	360	10	300	310	0.7197	0.6243-0.8151	0.7248	0.6269-0.8228	0.7704	0.6814-0.8595	0.7807	0.6903-0.8712				
	Cobas-OT	ASCUS higher	2228	1182	2218	2218	0.708	0.674-0.742	0.7113	0.6775-0.7451	0.7473	0.7149-0.7797	0.7497	0.7175-0.7819				
		ASC-H higher	2709	701	2358	2620	0.7346	0.7038-0.7653	0.7508	0.721-0.7805	0.7931	0.7653-0.8209	0.8034	0.7765-0.8304				
		LSIL higher	2792	618	2324	2905	0.7622	0.7334-0.791	0.7767	0.7488-0.8046	0.8253	0.7999-0.8507	0.8332	0.8087-0.8578				
		HSIL higher	3313	97	3024	3108	0.6163	0.585-0.6475	0.6179	0.5866-0.6491	0.6235	0.5922-0.6547	0.6245	0.5932-0.6557				
	Cobas-16	ASCUS higher	88	78	88	78	0.7889	0.629-0.9488	0.7855	0.6243-0.9467	0.7491	0.576-0.9222	0.7474	0.574-0.9208				
		ASC-H higher	107	59	118	118	0.861	0.7552-0.9669	0.8601	0.7541-0.9662	0.854	0.7435-0.9645	0.8531	0.7425-0.9638				
		LSIL higher	118	48	96	96	0.6264	0.4422-0.8106	0.6576	0.4792-0.836	0.6318	0.4477-0.8159	0.6603	0.4827-0.838				
		HSIL higher	143	23	165	161	0.8139	0.7046-0.9231	0.8435	0.7431-0.9439	0.8204	0.7125-0.9283	0.8463	0.7459-0.9467				

Supplemental Table 5. Comparison of AUCs between different platforms by T-test.

	ACD				POS			
	**HC2**	**E6E7**	**Cobas-OT**	**Cobas-16**	**HC2**	**E6E7**	**Cobas-OT**	**Cobas-16**
E6E7	0.000				0.000			
Cobas-OT	0.000	0.003			0.000	0.696		
Cobas-16	0.016	0.150	0.667		0.004	0.045	0.215	
ACD-POS	0.000	0.000	0.000	0.077				

P value less than 0.05 was recognized as significantly different.

Supplemental Table 6. Comparison of AUCs between HC2 models of different variable factor combinations by T-test.

	ACD			POS		
	**Factor (VL+Age)**	**Factor(VL+BV)**	**Factor(VL+BV+Age)**	**Factor (VL+Age)**	**Factor(VL+BV)**	**Factor(VL+BV+Age)**
**Factor (VL)**	0.028	0.010	0.460	0.038	0.008	0.202
**Factor (VL+Age)**		0.020	0.016		0.005	0.001
**Factor(VL+BV)**			0.927			0.065

P value less than 0.05 was recognized as significantly different.

Supplemental Table 7. Comparison of AUC values between different ML methods of HC2 ACD by T-test.

Data sets	ML method	xgboost	Random forrest	SVM	Logistic regression	Neural net
ACD	Decision tree	0.015	0.036	0.025	0.024	0.020
	xgboost		0.005	0.054	0.052	0.049
	Random forrest			0.870	0.780	0.007
	SVM				0.087	0.107
	Logistic regression					0.110
POS	Decision tree	0.199	0.002	0.096	0.096	0.087
	xgboost		0.314	0.089	0.065	0.019
	Random forrest			0.816	0.975	0.007
	SVM					0.179
	Logistic regression					0.159

P value less than 0.05 was recognized as significantly different.

Supplemental Table 8. Different HPV RLU values and corresponding cervical precancerous lesions

RLU	NILM	ASCUS	ASC-H	LSIL	HSIL	Total_number
>0,<=1	12633 (96.010%)	458 (3.481%)	6 (0.046%)	61 (0.464%)	0 (0.000%)	13158
>1,<=10	7994 (88.586%)	701 (7.768%)	49 (0.543%)	250 (2.770%)	30 (0.332%)	9024
>10,<=100	3450 (74.305%)	651 (14.021%)	153 (3.295%)	272 (5.858%)	117 (2.520%)	4643
>100,<=1000	1549 (44.018%)	793 (22.535%)	226 (6.422%)	639 (18.159%)	312 (8.866%)	3519
>1000	250 (15.528%)	268 (16.646%)	58 (3.602%)	817 (50.745%)	217 (13.478%)	1610

Supplemental Figure 1. All and positive cases distribution of all three datasets at different cytology stages. *: Number of HPV positive patient in each stage divided by number of HPV positive patient in ACD; +: Number of HPV positive patient in each stage divided by number of ACD.

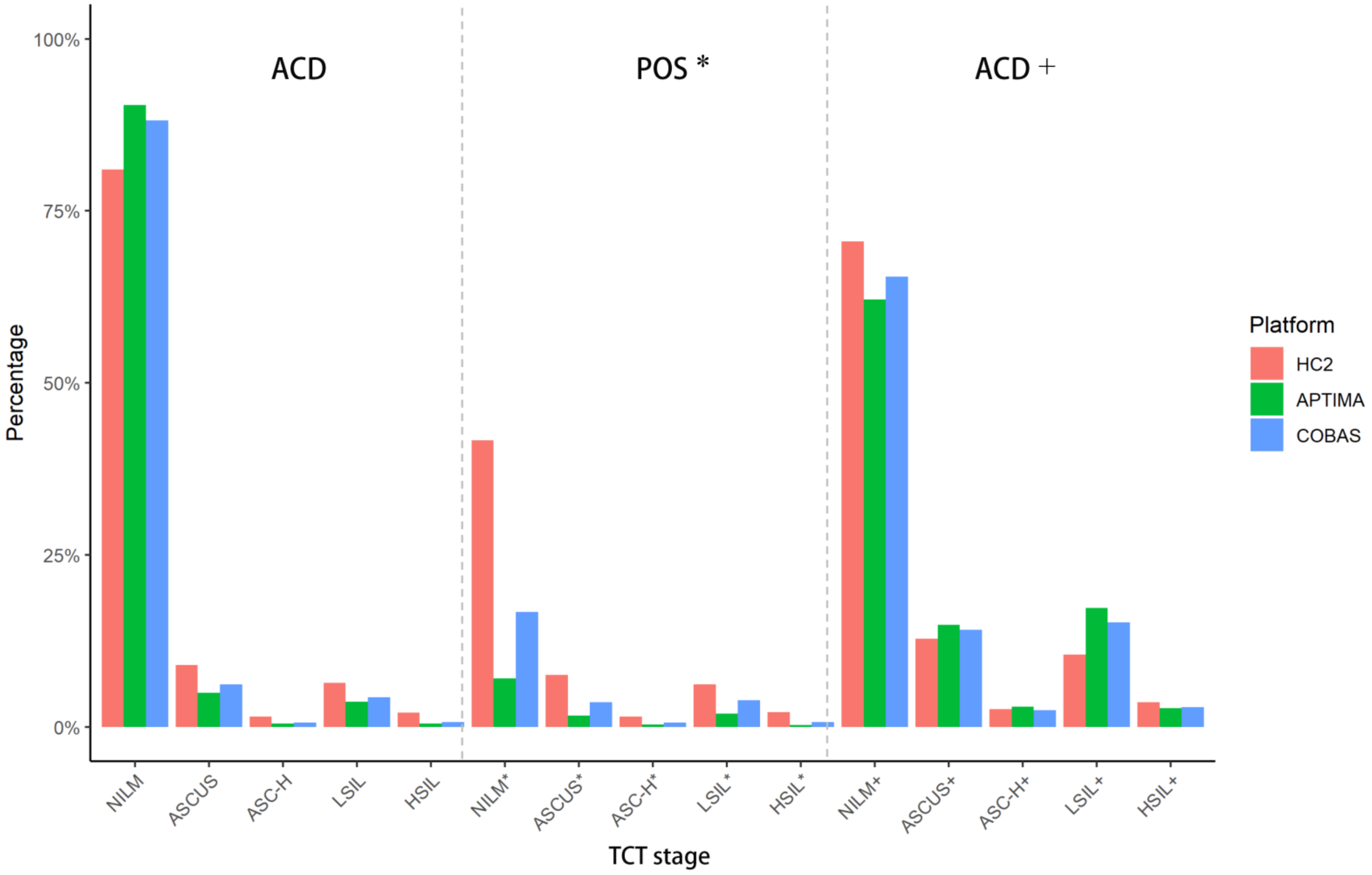

Supplemental Figure 2. Distribution of viral load value with cervical lesion stages of the three platform positive data sets. a. HC2. b. E6E7. c. Cobas HPV OT. d. Cobas HPV16. e. Cobas HPV18.

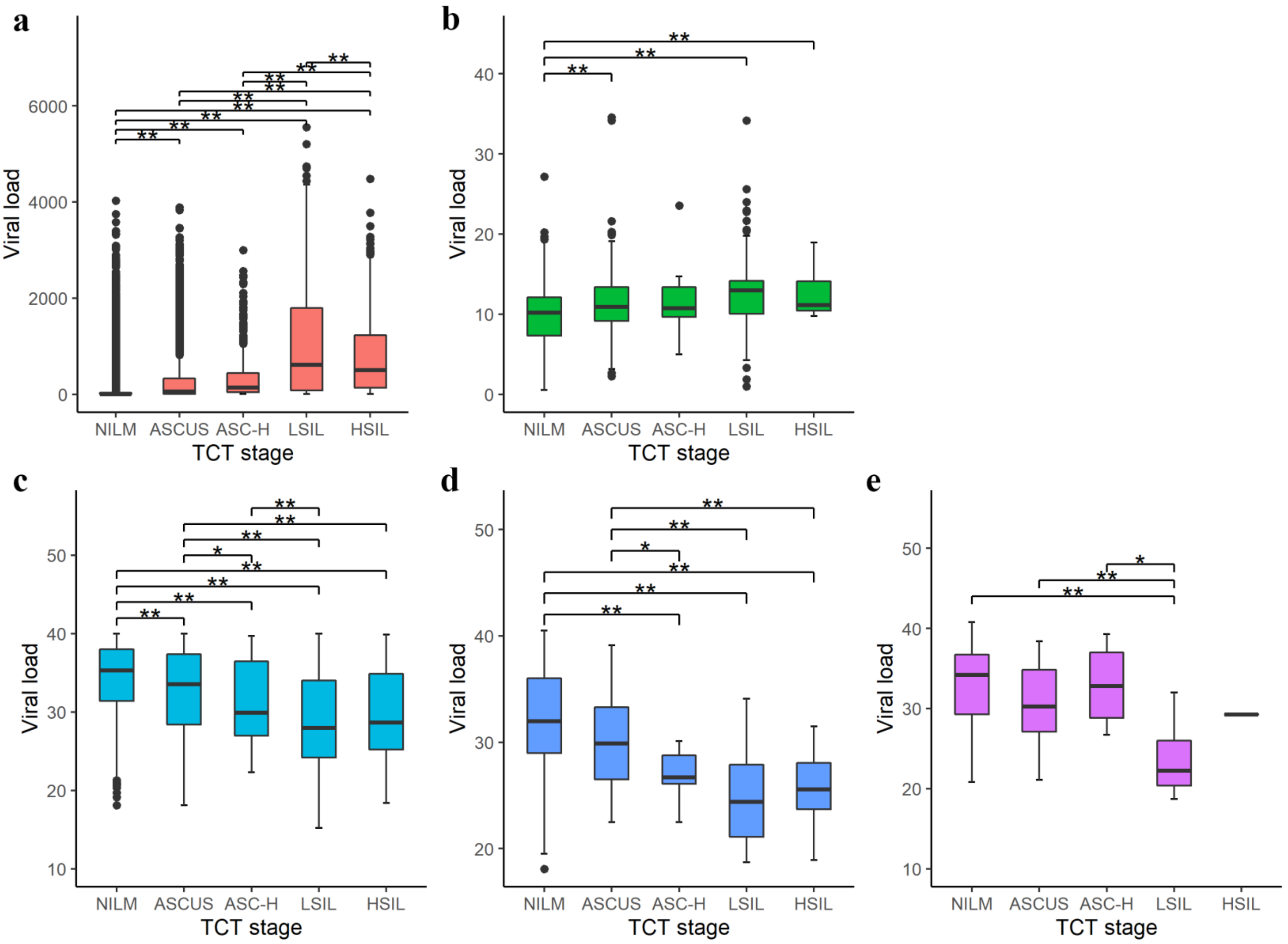

Supplement: Supplementary file 1 — Additional file 1. Other details of this study. [file 12985_2022_1908_MOESM1_ESM.pdf]
